# Reducing Noise Induced by Cardiac Pulsatility in Brain Maps of *R*_2_* and Magnetic Susceptibility Using Tailored k‐space Sampling

**DOI:** 10.1002/nbm.70305

**Published:** 2026-05-10

**Authors:** Quentin Raynaud, Thomas Dardano, Rita Oliveira, Giulia Di Domenicantonio, Tobias Kober, Christopher W. Roy, Ruud B. van Heeswijk, Antoine Lutti

**Affiliations:** ^1^ Laboratory for Research in Neuroimaging, Department for Clinical Neuroscience Lausanne University Hospital and University of Lausanne Lausanne Switzerland; ^2^ Department of Diagnostic and Interventional Radiology Lausanne University Hospital and University of Lausanne Lausanne Switzerland; ^3^ Advanced Clinical Imaging Technology Siemens Healthineers International AG Lausanne Switzerland; ^4^ LTS5, École Polytechnique Fédérale de Lausanne (EPFL) Lausanne Switzerland

**Keywords:** brain, cardiac‐induced noise, MRI relaxometry, physiological noise, QSM, quantitative MRI, *R*_2_*

## Abstract

Maps of the transverse relaxation rate *R*_2_* and magnetic susceptibility (*χ*) are computed from gradient‐echo data and are sensitive to signal instabilities induced by cardiac pulsation. Here, we introduce two k‐space sampling strategies that aim to mitigate the impact of cardiac‐induced noise in brain maps of *R*_2_* and *χ*.

The proposed strategies are based on the higher level of cardiac‐induced noise near the k‐space centre compared to the periphery. Using CArtesian trajectory with Spiral PRofile (CASPR), the first strategy allows for the acquisition of a specific number of averages at each k‐space location, derived from the local level of cardiac‐induced noise. The second strategy uses cardiac triggering to synchronize the acquisition near the k‐space centre with the cardiac cycle in real time. We compared the variability across four repetitions of *R*_2_* and *χ* maps computed from data acquired using both strategies and with a standard linear trajectory.

Data were acquired in 10 healthy volunteers. Compared to linear trajectory, CASPR reduced the variability of *R*_2_* and *χ* maps across repetitions by 22% and 16% across the whole brain, reaching over 30% in inferior brain regions, for a 14% increase in scan time. CASPR also reduced the level of aliasing artefacts from pulsating blood vessels. Cardiac triggering did not reduce the variability of *R*_2_* or *χ* maps.

CASPR can be designed to mitigate cardiac‐induced noise in brain maps of the MRI parameters *R*_2_* and *χ*. Synchronization of data acquisition with the cardiac cycle did not reduce the level of cardiac‐induced noise.

## Graphical abstract

With linear k‐space sampling trajectories, cardiac pulsation leads to MRI signal instabilities that increase the variability (standard deviation, SD) of *R*_2_* and QSM maps across repetitions. Here, we propose alternative trajectories that aim to mitigate the impact of cardiac‐induced noise in brain maps of *R*_2_* and QSM. In particular, the proposed CASPR trajectory reduces the SD of *R*_2_* and QSM maps by 22% and 16% across repetitions.

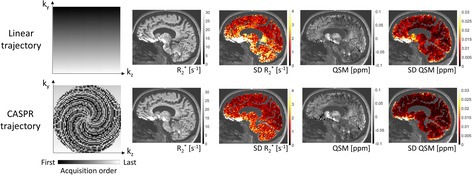

## 1 Introduction

MRI relaxometry enables the noninvasive investigation of microscopic changes in brain tissue in patient populations [1, 2]. Estimates of the transverse relaxation rate *R*_2_* (= 1/*T*_2_*) [3] and magnetic susceptibility (χ) [4] are biomarkers of iron and myelin concentration within brain tissue [2, 5], and provide means to monitor the evolution of neurological diseases such as Parkinson's disease [6], Alzheimer's disease [7], and multiple sclerosis [8].

Cardiac pulsation leads to a systolic blood pressure wave that travels to the brain and generates periodic instabilities of the MRI signal through head motion [9], brain tissue deformation [10], blood flow [11], cerebrospinal fluid flow [12], changes in the tissue's O_2_/CO_2_ concentration [13], blood vessel pulsatile motion [14] and other effects [15]. These instabilities are most prominent in inferior and highly vascularized brain regions such as the orbitofrontal cortex [16], brainstem [17], cerebellum [18] and periventricular regions [19]. Changes of the B_0_‐field [20], laminar flow [21] and motion [22] are coherent across an image voxel and lead to a net phase shift of the signal. Turbulent flow [23] and intra‐voxel B_0_ inhomogeneities [24] are incoherent across an image voxel and affect the magnitude of the signal. Cardiac pulsation leads to aliasing artefacts [25] and enhances the noise level in the acquired data, reducing sensitivity to brain change in neuroscience studies [26, 27].

Acquisition of MRI relaxometry data requires several minutes and the effects of cardiac‐induced signal instabilities are challenging to reduce with postprocessing techniques. Instead, prospective strategies have been proposed to ensure that cardiac‐induced noise remains incoherent along the spatial [28] or temporal [25] dimensions of the multiecho GRE data. These passive scrambling methods, which aim to mitigate the effect of cardiac‐induced noise rather than reduce its amplitude, remove aliasing in *R*_2_* and χ maps and improve their reproducibility by ~25% [25]. Strategies that reduce the level of cardiac‐induced noise in GRE data target in‐flowing arterial blood [29, 30]. However, these techniques only address a subset of the physiological effects that contribute to cardiac‐induce noise. They also require tailored radiofrequency (RF) saturation pulses or gradient waveforms that reduce the efficiency of data acquisition. Instead, reduction of cardiac‐induced noise in GRE data may be achieved from alternative k‐space sampling trajectories: Keyhole imaging or PROPELLER acquires the centre of k‐space multiple times to resolve dynamic effects such as blood flow [31] or BOLD signals [32] and are tailored to increase SNR or reduce motion [33]. In nonbrain imaging, dynamic keyhole acquisition can be used to resolve and mitigate breathing effects or motion [34, 35]. The adaptation of these methods towards the reduction of cardiac‐induced noise in GRE data remains unexplored.

In this study, we introduce two data acquisition strategies that aim to reduce the level of cardiac‐induced noise in brain relaxometry data. These strategies were derived from a recent characterization of cardiac‐induced noise in multiecho GRE data [27]. The first strategy (CArtesian trajectory with Spiral PRofile ordering, CASPR) [35], acquires a specific number of averages at each k‐space location, set according to the local level of cardiac‐induced noise. The second strategy, inspired by the use of cardiac gating for diffusion [36] and functional MRI [37], synchronizes data acquisition near the k‐space centre with cardiac pulsation to acquire the most sensitive data during the most stable part of the cardiac cycle. Following the optimization of both strategies using numerical simulations, we evaluated their ability to reduce the level of cardiac‐induced noise in repeated multiecho GRE data acquired in vivo.

## 2 Methods

The proposed strategies to reduce the level of cardiac‐induced noise were derived from a recent characterization of cardiac‐induced noise in GRE data [27]. In that study, low‐resolution 3D multiecho GRE data were acquired continuously for 1 h in five participants. The participants' cardiac pulsation was recorded simultaneously. To resolve the effect of cardiac pulsation on *R*_2_* maps, the data were reordered according to the phase of the cardiac cycle at the time of its acquisition, leading to 5D datasets with three spatial dimensions, one dimension for the echo time and one dimension for the phase of the cardiac cycle. By analogy with other models of physiological noise [38], cardiac‐induced noise was modelled by second‐order Fourier series decomposition of the change of the 5D data along its cardiac cycle dimension [27]. This enabled the estimation of the amplitude of cardiac‐induced noise at each k‐space location and echo time.

Here, we used the cardiac‐induced noise modelled from the 5D datasets to conduct numerical simulations of candidate k‐space sampling strategies and to evaluate their ability to reduce cardiac‐induced noise in multiecho GRE data.

### 2.1 Strategy 1: CArtesian Trajectory With Spiral PRofile (CASPR)

**FIGURE 1:**
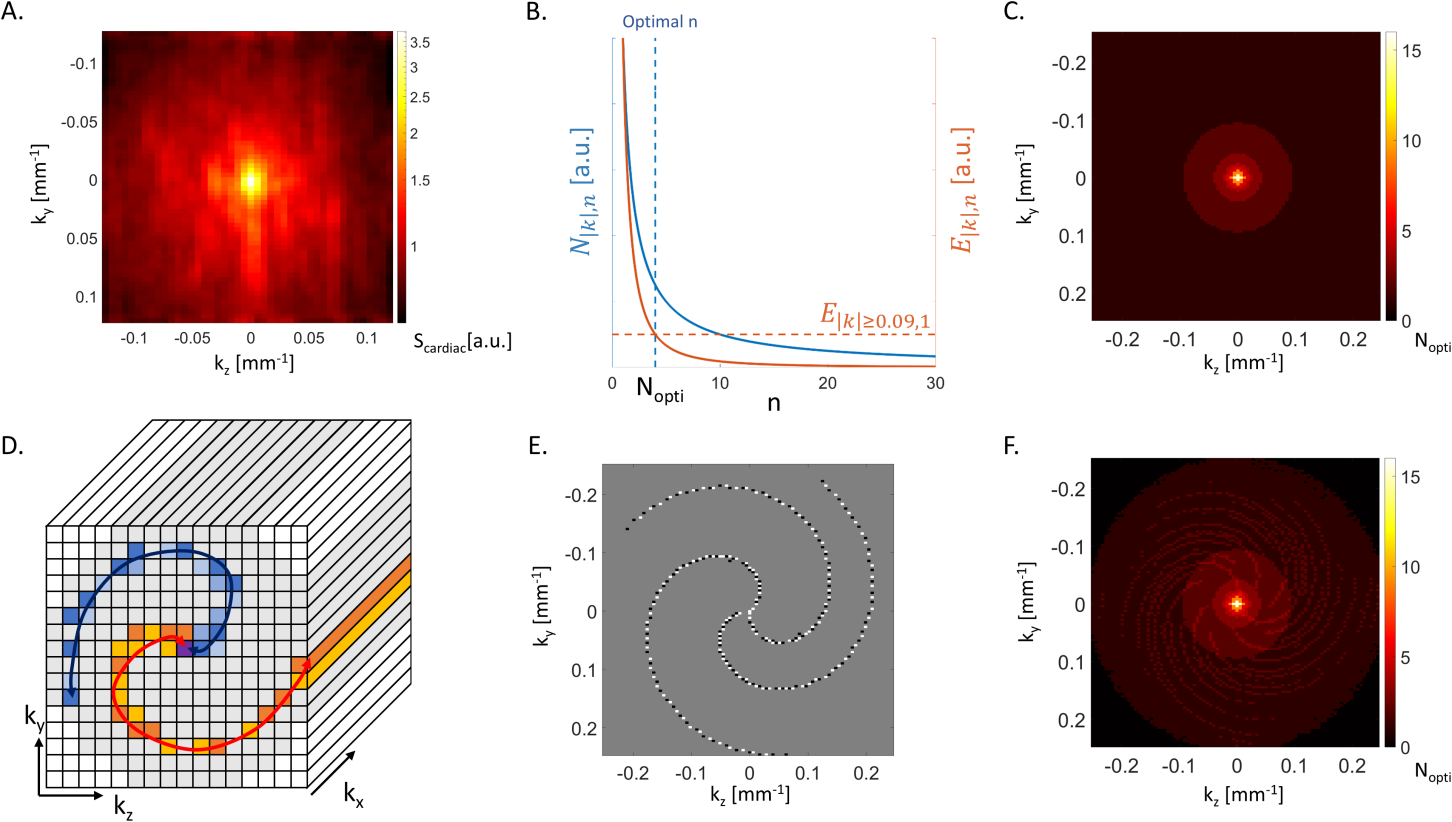
Cardiac noise and the proposed k‐space trajectory. (A) Distribution of the amplitude of the modelled cardiac‐induced noise $S_{\text{cardiac}}$ along the two phase‐encoding directions ($k_{y},k_{z}$) of a 3D GRE sequence [27]. (B) Effective noise level ($N_{\left|k\right|,n}$) and averaging efficiency ($E_{\left|k\right|,n}$) as a function of the number of averages *n*. The optimal number of averages *N*_*opti*_ at a given k‐space location is reached when the averaging efficiency ($E_{\left|k\right|,n}$) equals that of a point at the k‐space periphery dominated by thermal noise. (C) Target k‐space distribution of *N*_*opti*_. (D) Graphical representation of the CASPR trajectory in 3D k‐space. For each spiral arm, the dark/light data points are acquired on the Cartesian grid in the outward/inward direction. (E) First three spiral arms of the optimal CASPR trajectory. The white/black data points are acquired on the Cartesian grid in the outward/inward direction. (F) Distribution of *N*_*opti*_ achieved with the optimal CASPR trajectory. The k‐space corners (in black) are acquired linearly at the end of the CASPR acquisition.

The amplitude of the modelled cardiac‐induced noise depends primarily on the radial distance $\left|k\right|$ to the centre of k‐space along the two phase‐encoding directions ($k_{y},k_{z}$) of a 3D GRE sequence (Figure 1A): 50%–60% of cardiac‐induced noise is located near the k‐space centre ($\left|k\right|$ < 0.074 mm ^− 1^) [27]. With Strategy 1, the number of averages at a distance $\left|k\right|$ from the k‐space centre is set according to the local level of cardiac‐induced noise. The effective noise level in the data is:

(1) $$N_{\left|k\right|,n}=\mathrm{var}\left(S_{\text{cardiac}}\right)/n,$$

where n is the number of averages and $\mathrm{var}\left(S_{\text{cardiac}}\right)$ is the variance of the modelled cardiac‐induced signal fluctuations across the cardiac cycle [27].

The averaging efficiency, that is, the reduction in effective noise level from the acquisition of one additional average, is given by:

(2) $$E_{\left|k\right|,n}=N_{\left|k\right|,n+1}-N_{\left|k\right|,n}=N_{\left|k\right|,1}\left(\frac{1}{n+1}-\frac{1}{n}\right),$$

where $N_{\left|k\right|,1}=\mathrm{var}\left(S_{\text{cardiac}}\right)$ is the effective noise level when only one sample is acquired. Equation 2 shows that averaging efficiency decreases with an increasing number of averages (Figure 1B). Enforcing a uniform effective noise level across k‐space (Equation (1)) would require a high number of averages near the k‐space centre. Because efficiency decreases with the number of averages, this would lead to a prohibitive extension of scan time. Instead, we set the optimal number of averages $N_{\text{opti}}$ so that the averaging efficiency is at least equal to that of the k‐space periphery ($\left|k\right|\geq 0.09$ mm ^− 1^), where thermal noise is the dominant source of noise and only one sample is acquired (n=1):

(3) $$E_{\left|k\right|<0.09,N_{\text{opti}}}=N_{\left|k\right|,1}\left(\frac{1}{N_{\text{opti}}+1}-\frac{1}{N_{\text{opti}}}\right)\geq -\frac{1}{2}N_{\left|k\right|\geq 0.09,n=1},$$

Figure 1C shows the distribution of $N_{\text{opti}}$ in the 2D plane of the two phase‐encoding directions ($k_{y},k_{z}$). This distribution, which peaks at $N_{\text{opti}}\sim 15$ near the k‐space centre, was achieved using a CArtesian trajectory with Spiral PRofile (CASPR) [35, 39]. CASPR trajectories acquire data on spiral arms projected on a Cartesian grid along the two phase encoding directions (Figure 1D). For each combination of ($k_{y},k_{z}$) along the spiral arms, k‐space sampling is conducted linearly along the readout direction, for all echoes. Each spiral arm starts at the k‐space center (black) and then goes outward (orange) and inward (yellow). The next spiral arm is rotated by the golden angle (blue) [40, 41]. CASPR trajectories with different number of points per arms (10–200), number of arms (10–200) and sampling density (0.1–1.75) were simulated. The CASPR trajectory that led to the closest match with the target number of averages consisted of 120 spiral arms with 100 points each (Figure 1E). The spatial frequencies in the corners of k‐space (black regions of Figure 1F), not acquired with the CASPR trajectory, are acquired using a linear scheme at the end of the acquisition.

While CASPR is also used with non‐Cartesian trajectories [40], the proposed implementation samples k‐space data on a 3D Cartesian grid: The ($k_{y},k_{z}$) positions are defined on a Cartesian grid and sampling along the readout direction is conducted using a Cartesian linear trajectory, using trapezoidal gradient waveforms. As a result, no data interpolation or nonuniform Fourier transformation is required prior to image reconstruction.

### 2.2 Strategy 2: Cardiac‐Triggered Sampling

Strategy 2 proposes to use cardiac triggering to enforce data acquisition during the part of the cardiac cycle where the GRE signal is most stable. Here, cardiac triggering locks the data acquisition to a fixed phase of the cardiac cycle at specific k‐space locations only: Preceding and subsequent peripheral k‐space locations are sampled without locking. To limit the resulting extension of scan time, cardiac triggering was only applied near the k‐space centre (|*k*| < 0.125 mm^−1^), where most cardiac‐induced noise is present [27].

**FIGURE 2:**
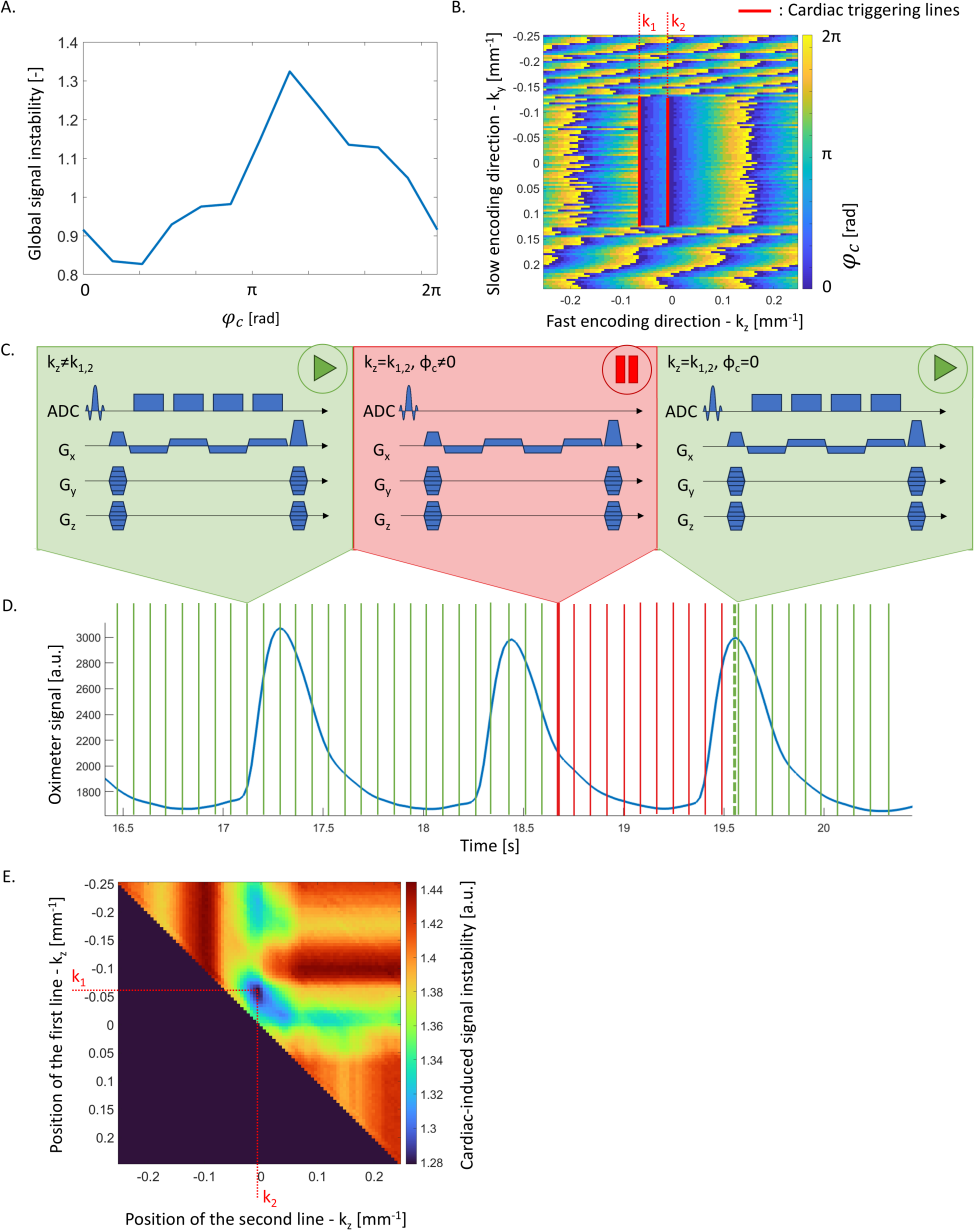
Proposed cardiac‐triggered sampling. (A) Global signal instability induced by cardiac pulsation. (B) Phase of the cardiac cycle at the time of acquisition of the data with the proposed cardiac‐triggered linear trajectory. The fast ($k_{z}$)/slow ($k_{y}$) phase‐encoding directions of the 3D GRE sequences are displayed horizontally/vertically. K‐space sampling was started from the top‐left corner. The red cardiac triggering lines enforce acquisition of the k‐space centre data during the most stable part of the cardiac cycle ($0<\varphi_{c}<\frac{\pi }{2}$). (C) Pulse sequence diagram of the cardiac triggered sampling. At $k_{z}=k_{1}$ or $k_{z}=k_{2}$, analogue‐to‐digital conversion (ADC) is disabled until the detection of the next peak in the pulse oximeter signal. (E) Cardiac‐induced noise level as a function of the position of the two cardiac triggering lines $k_{1}$ and $k_{2}$.

We conducted numerical simulations to identify an implementation of cardiac triggering that leads to maximal reduction of cardiac‐induced noise in the acquired data. From the 5D model of cardiac‐induced noise, we computed the first order derivative along the direction of the cardiac cycle, then averaged it along the readout ($k_{x}$) and echo time directions, resulting in a 3D matrix containing the derivatives of the modelled cardiac‐induced noise along the direction of the cardiac cycle, for each ($k_{y},k_{z}$) coordinate of the phase‐encoding plane. This matrix was used as a fingerprint of signal instability induced by cardiac pulsation, for each ($k_{y},k_{z}$) coordinate and phase of the cardiac cycle. The global signal instability is lower during the first quarter of the cardiac cycle ($0<\varphi_{c}<\frac{\pi }{2}$) (Figure 2A).

Simulation of data acquisition consisted of selecting one value of cardiac‐induced signal instability for each ($k_{y},k_{z}$) coordinate, depending on the sequence timing and the cardiac period T, that is, the time required to span the 2π range of the cardiac phase dimension of the matrix of cardiac‐induced signal instability. The simulated MRI acquisition had a repetition time (TR) of 40 ms and a matrix size of 88 along the fast phase‐encoding direction ($k_{z}$). K‐space sampling was started from one corner of k‐space, as a standard linear trajectory, with no locking to the cardiac cycle (Figure 2B). For each consecutive k‐space coordinate in the slow phase‐encoding direction ($k_{y}$), data were acquired linearly along the fast‐phase‐encoding direction ($k_{z}$), at a rate of 1/TR = 25 Hz. As a result, the phase of the cardiac cycle at the time of acquisition of the data varied smoothly along $k_{z}$. When the k‐space centre was reached (|*k*| < 0.125 mm ^− 1^), data acquisition was locked to the cardiac cycle by means of two cardiac triggering lines aligned along the slow phase‐encoding direction $k_{y}$ (Figure 2B). Upon reaching these lines, data acquisition was paused, maintaining RF excitation to preserve the steady state of the MRI signal (Figure 2C) [42]. K‐space sampling was resumed when the next peak of the pulse wave was detected ($\varphi_{c}=0$) (Figure 2D). As a result, acquisition of the subsequent data points took place during the most stable period of the cardiac cycle (i.e., the first quarter after systole). A global estimate of signal instability was computed by summing the selected cardiac‐induced signal instabilities across all ($k_{y},k_{z}$) coordinates. The simulations were repeated, varying the cardiac period T from 600 to 1000 ms in steps of 10 ms.

The numerical simulations were conducted for every possible position of the two cardiac triggering lines. The optimal position of the cardiac triggering lines, identified from the minimum global signal instability induced by cardiac pulsation, was $k_{1}=-0.0625$ mm^−1^ and $k_{2}=-0.0057$ mm^−1^ along the fast‐phase‐encoding direction (Figure 2E).

### 2.3 MRI Data Acquisition and Analyses

To assess the reduction in cardiac‐induced noise from the two proposed strategies, data were acquired in 10 healthy volunteers (7 females, 27 ± 7 years old), with a 3 T MRI scanner (Magnetom Prisma, Siemens Healthineers, Forchheim, Germany) and a 64‐channel head–neck coil. The participants' cardiac pulsation was recorded using a pulse‐oximeter attached to their finger. To ensure optimal quality of the pulse‐oximeter signal, participants were offered a blanket and a hot water bottle was placed near their hand. The study was approved by the local ethics committee (CER‐VD) and all participants gave their written informed consent prior to participation.

#### 2.3.1 MRI Protocol

MRI data were acquired with a custom‐made 3D multiecho FLASH sequence capable of k‐space sampling with a standard linear Cartesian trajectory as well as the proposed CASPR trajectory and cardiac triggering. 15 echo images were acquired after radiofrequency (RF) excitation with echo times TE = 2.34–35.10 ms. The repetition time was 40 ms, the RF excitation flip angle was 16° and image resolution was 2 × 2 × 2 mm^3^ (matrix size 128 × 128 × 88). GRAPPA [43] was used with acceleration factor of 2 and 24 reference lines. The scan time of each repetition was 4:31 min for the standard linear trajectory, 5:09 min (+14%) for the CASPR trajectory and 5:22 min (+19 ± 1%) for the cardiac‐triggered sampling. Four repetitions were conducted for each sampling strategy to quantify the reproducibility of the data. The spatial resolution of the data was low to keep the total scan time reasonable. Additional MRI data were acquired on a single participant (male, 30 years old) with a higher image resolution of 1.2 × 1.2 × 1.2 mm^3^ (matrix size 208 × 192 × 144), representative of relaxometry protocols used in neuroscience studies [44]. The other acquisition parameters were unchanged. Three repetitions of the high‐resolution protocol were conducted with standard linear trajectory (scan time 10:28 min) and CASPR trajectory (scan time 13:30 min, +29%).

All acquisition protocols also included an MP‐RAGE [45] image for segmentation and anatomical reference (1 mm^3^ resolution, TR/TE = 2000/2.39 ms, inversion time = 920 ms, GRAPPA [43] acceleration factor 2 with 24 reference lines, RF excitation angle = 9°, acquisition time 4:16 min). Two 3D FLASH datasets were acquired with the head‐ and body coils for signal reception (4 × 4 × 4 mm^3^ image resolution, TR/TE = 5.72 ms/2.34 ms, excitation flip angle = 6°, acquisition time 16 s) and used subsequently for the computation of the coil sensitivity maps [46].

#### 2.3.2 Image Reconstruction

Brain images were reconstructed offline using Matlab (Version 2022a, The MathWorks, Natick, MA, USA). Coil sensitivity maps were computed as the ratio of the (4 mm) [3] resolution data acquired with the head and body coils [46, 47]. For CASPR trajectory, the multiple samples acquired at each k‐space location were averaged [48]. Coil‐specific images were reconstructed with GRAPPA [43] (https://github.com/mchiew/grappa‐tools) and combined by performing a SENSE [47] reconstruction with an acceleration factor of 1.

#### 2.3.3 Computation and Analysis of the qMRI Maps

##### 2.3.3.1 Relaxometry and QSM

*R*_2_* maps were computed voxel‐wise from multiecho images on each repetition using a regression of the log signal with the corresponding echo times [49]. The noise level on the *R*_2_* estimates was calculated as the root‐mean‐squared error (RMSE) between the MR signal and the *R*_2_* fit.

Maps of magnetic susceptibility (χ) were generated from the phase of the MR data of each repetition using bespoke scripts adapted from https://github.com/fil‐physics/MPM_QSM and following the ISMRM consensus guidelines [50]. The phase maps were unwrapped using ROMEO [51] with an additional correction for linear phase offsets induced by bipolar readouts [52]. Brain masks were generated with BET from FSL [53] and underwent subsequent refinement through multiplication with a phase‐quality‐based mask obtained during the ROMEO unwrapping process, and any holes present within the resulting mask were subsequently filled. Removal of the background field was conducted with the Projection onto Dipole Fields algorithm [54] available in the SEPIA toolbox [55]. Finally, dipole inversion was computed using the STAR‐QSM algorithm [56] provided in the SEPIA toolbox, using the entire brain as a reference. In one subject, an interhemispheric calcification was manually masked out and dipole inversion was conducted using this adjusted mask.

##### 2.3.3.2 Image Segmentation and Statistical Analysis

Image coregistration and segmentation were conducted using Statistical Parametric Mapping (SPM12, Wellcome Centre for Human Neuroimaging, London, UK). The MP‐RAGE images were segmented into maps of grey and white matter probabilities using Unified Segmentation [57]. Whole‐brain masks were computed from the grey and white matter segments and included voxels with a combined probability of 0.9 or above. As described in Lutti et al. [44], occipital, frontal, parietal and temporal grey matter regions of interest (ROIs) were computed from the grey matter maximum probability labels computed in the ‘MICCAI 2012 Grand Challenge and Workshop on Multi‐Atlas Labeling’ (https://masi.vuse.vanderbilt.edu/workshop2012/index.php/Challenge_Details), using MRI scans from the OASIS project (http://www.oasis‐brains.org/) and labelled data provided by Neuromorphometrics Inc. (http://neuromorphometrics.com/) under academic subscription. White matter ROIs of the corticospinal tract, inferior longitudinal fasciculus and optic radiations were computed from the JHU DTI‐based atlases (https://identifiers.org/neurovault.collection:264) [58, 59, 60]. Additional brainstem and cerebellum ROIs were computed from the corresponding atlas labels and combined grey matter and white matter probabilities.

Cardiac pulsation leads to exponential‐like and nonexponential effects on the magnitude of the MRI signal [27]. The level of cardiac‐induced noise in the magnitude of the MRI data were therefore assessed from the estimates of *R*_2_* and RMSE. The level of cardiac‐induced noise in the phase of the MRI was assessed from the χ estimates alone: the noise level on these estimates could not be computed from the code used for the generation of the χ maps.

Because cardiac‐induced noise increases the variability of *R*_2_*, RMSE and χ estimates [25, 27], the standard deviation (SD) of these metrics across repetitions was used in the statistical comparisons of the proposed acquisition strategies. Additionally, an increase of the mean RMSE across repetitions has been reported as an indicator of the successful mitigation of the exponential‐like effects of cardiac pulsation on the signal magnitude [25]. Statistical comparisons of the proposed acquisition strategies therefore also included the mean RMSE. We conducted Shapiro–Wilk tests of the normality of the distributions of the mean and variability of the *R*_2_*, RMSE and χ estimates across participants. As significant deviations from normality were observed, these comparisons were conducted using two‐sided Wilcoxon signed‐rank of regional averages.

## 3 Results

**FIGURE 3:**
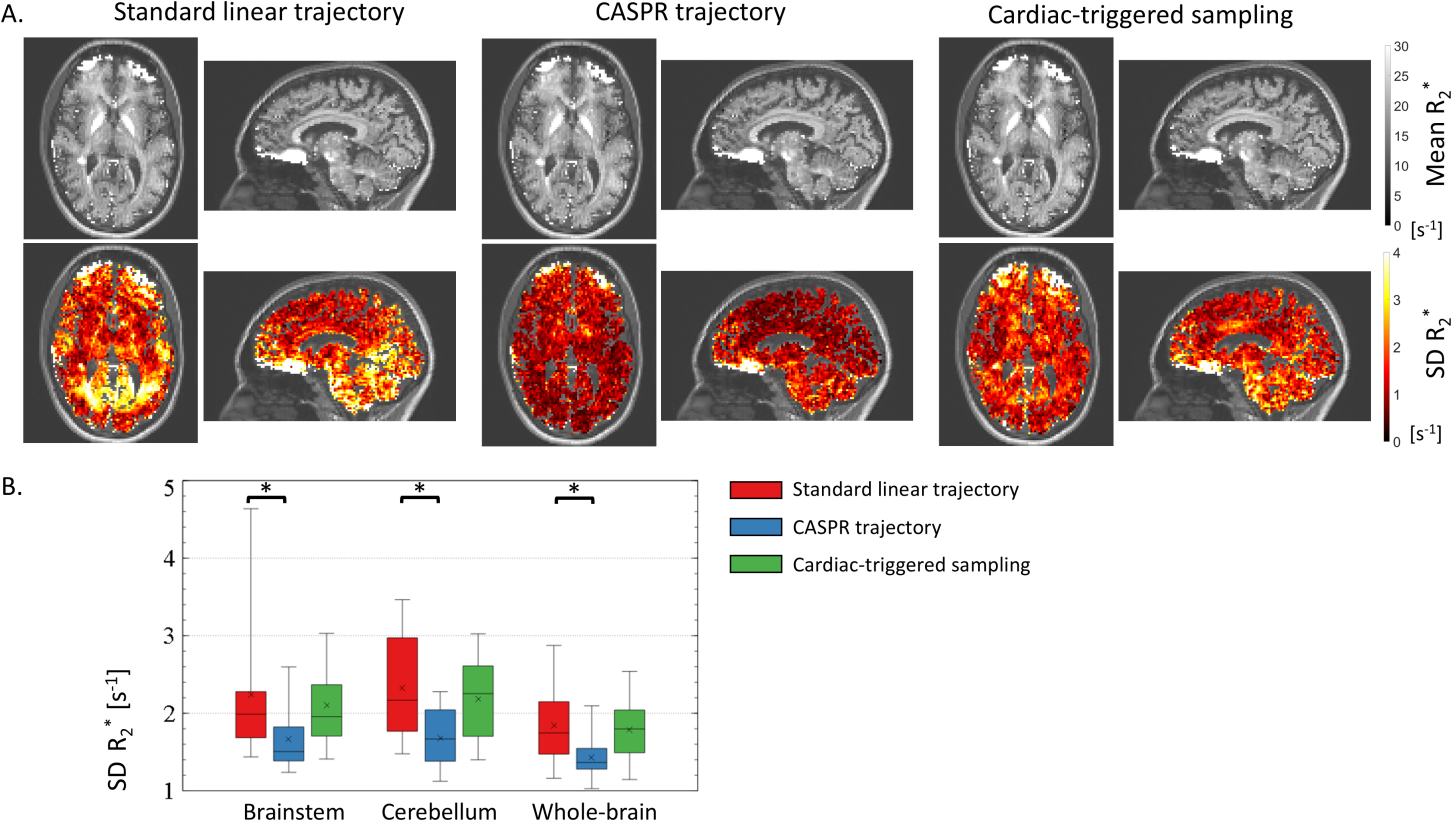
(A) Example maps of the mean and standard deviation (SD) of *R*_2_* across repetitions, computed from data acquired with the standard linear trajectory, CASPR trajectory and cardiac triggered sampling. To improve visibility, the presented maps were masked using a whole‐brain mask including grey and white matter voxels (see Section 2.3) and overlaid on the MPRAGE image. (B) Regional estimates of the SD of *R*_2_* across repetitions in the brainstem, cerebellum and whole brain, across all 10 healthy volunteers.

Across the 10 participants, systematic differences in *R*_2_* estimates between the CASPR trajectory/cardiac triggering and the reference standard linear trajectory are −0.15 and −0.08 s^−1^ respectively, that is, less than 1% of typical *R*_2_* values in the brain parenchyma (*R*_2_* ~ 20 s^−1^) (Supplementary Material S1). With the standard linear trajectory, the SD of *R*_2_* across repetitions reaches up to 4 s^−1^ in inferior brain regions (Figure 3A). With the CASPR trajectory, maps of the SD of *R*_2_* are more spatially uniform and the regional averages are reduced by 26/28/22% in the brainstem/cerebellum/whole‐brain compared to the standard linear trajectory (Figure 3, *p* ≤ 0.020). A reduction of the SD of *R*_2_* is present across all brain regions, reaching over 30% in inferior brain regions such as the occipital lobes, optic radiations and inferior longitudinal fasciculus (Table 1). The smallest reduction of the SD of *R*_2_* was ~20% in the grey matter of frontal lobe. This is larger than the $\sqrt{1.14}\sim 6.7\%$ reduction in variability expected from the 14% additional samples of the CASPR trajectory if thermal noise dominated signal variance. With cardiac triggering, the SD of *R*_2_* across repetitions is reduced by 6/6/3% in the brainstem/cerebellum/whole‐brain respectively, for an increase in scan time of ~19% (Figure 3, *p* ≥ 0.49). This is less than the reduction in variability expected from data acquired with the standard linear trajectory with 19% more samples to improve SNR ($\sqrt{1.19\;}\sim 9.0\%$). Post hoc analyses were conducted to investigate the underwhelming reproducibility of the data acquired with cardiac triggering (see Supplementary Material S2).

**TABLE 1:** ROI‐average mean and variability of *R*_2_* estimates across repetitions (median [interquartile range]), computed from data acquired with the standard linear trajectory, CASPR trajectory and cardiac triggered sampling.

	ROI‐average *R*_2_* (s^−1^)			ROI‐average *R*_2_* SD (s^−1^)		
	Standard linear	CASPR	Cardiac triggered sampling	Standard linear	CASPR	Cardiac triggered sampling
Brainstem	20.50 [19.40, 21.78]	20.71 [19.54, 21.62]	20.54 [19.60, 21.32]	1.99 [1.67, 2.31]	1.51 [1.35, 1.86]	1.96 [1.64, 2.43]
Cerebellum	20.04 [19.46, 20.75]	20.40 [19.39, 20.64]	20.17 [19.72, 21.07]	2.17 [1.75, 3.17]	1.67 [1.35, 2.08]	2.25 [1.57, 2.68]
Whole brain	20.48 [19.34, 21.04]	20.54 [19.35, 21.51]	20.52 [19.37, 21.23]	1.75 [1.44, 2.18]	1.37 [1.26, 1.57]	1.80 [1.41, 2.09]
Occipital lobe	19.36 [18.13, 20.09]	19.28 [17.85, 20.35]	19.35 [18.18, 20.40]	1.63 [1.27, 2.19]	1.19 [0.91, 1.34]	1.63 [1.21, 2.07]
Frontal lobe	16.64 [16.00, 16.85]	16.57 [16.20, 16.98]	16.51 [16.07, 16.74]	1.33 [1.21, 1.74]	1.06 [0.93, 1.40]	1.38 [1.19, 1.92]
Parietal lobe	17.45 [16.40, 17.87]	17.48 [16.31, 17.91]	17.49 [16.35, 17.79]	1.31 [1.01, 1.70]	0.99 [0.90, 1.12]	1.33 [1.01, 1.46]
Temporal lobe	18.45 [17.18, 19.00]	18.47 [17.02, 19.09]	18.50 [17.10, 19.20]	1.67 [1.50, 2.30]	1.28 [1.04, 1.63]	1.76 [1.32, 1.96]
Corticospinal tract	19.96 [19.55, 20.14]	20.11 [19.75, 20.31]	19.97 [19.91, 20.26]	1.59 [1.31, 1.91]	1.22 [1.12, 1.46]	1.73 [1.22, 1.88]
Inferior longitudinal fasciculus	20.08 [19.64, 20.72]	20.42 [19.91, 21.24]	20.27 [19.66, 20.59]	1.41 [1.17, 2.09]	1.18 [0.87, 1.30]	1.46 [1.23, 1.70]
Optic radiations	20.79 [20.55, 21.25]	20.93 [20.80, 21.31]	21.03 [20.83, 21.32]	1.09 [0.93, 2.02]	0.88 [0.69, 1.01]	1.35 [0.88, 1.60]

**FIGURE 4:**
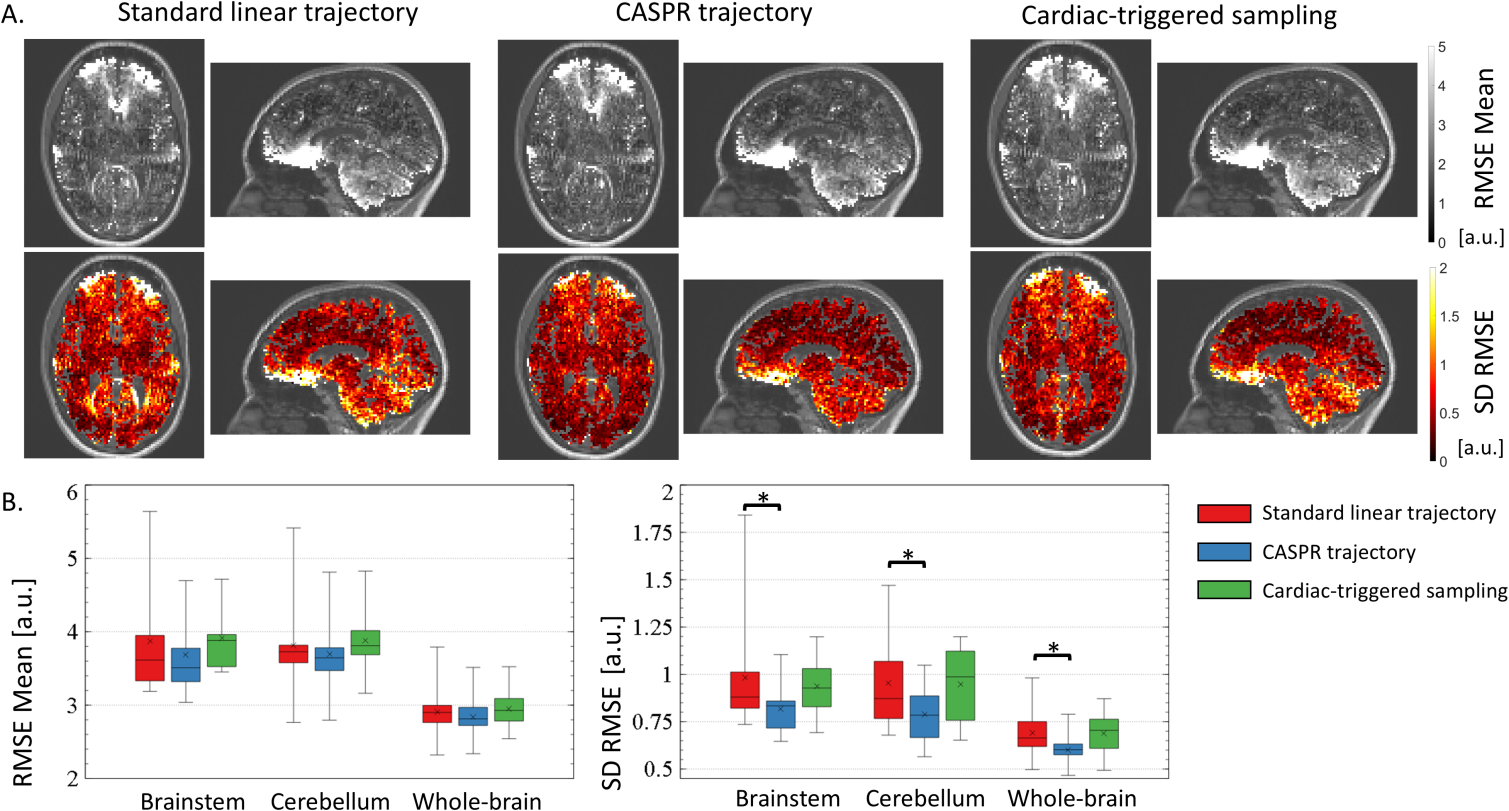
(A) Example maps of the mean and standard deviation (SD) of RMSE across repetitions, computed from data acquired with the standard linear trajectory, CASPR trajectory and cardiac triggered sampling. To improve visibility, the presented maps were masked using a whole‐brain mask including grey and white matter voxels (see Section 2.4.2) and overlaid on the MPRAGE image. (B) Regional estimates of the mean RMSE in the brainstem, cerebellum and whole brain, across all 10 healthy volunteers. (C) Regional estimates of the SD of RMSE in the brainstem, cerebellum and whole brain, across all 10 healthy volunteers.

The maps of the mean and SD of RMSE across repetitions appear largely comparable between the three sampling strategies (Table 2 and Figure 4A). Compared to standard linear trajectory, the mean RMSE is lower by 5%/3%/2% with the CASPR trajectory (*p* ≥ 0.06) and higher by 1%/2%/1% with cardiac triggering (*p* ≥ 0.16), in the brainstem/cerebellum/whole‐brain, respectively (Table 2 and Figure 4B). With the CASPR trajectory, the SD of RMSE across repetitions is lower by 17%/17%/13% in the brainstem/cerebellum/whole‐brain compared to standard linear trajectory (Figure 4B, *p* ≤ 0.019). With cardiac triggering, the SD of RMSE across repetitions is higher by 5%/1%/0% (Figure 4B, *p* ≥ 0.62).

**TABLE 2:** ROI‐average mean and variability of RMSE estimates across repetitions (median [interquartile range]), computed from data acquired with the standard linear trajectory, CASPR trajectory and cardiac triggered sampling.

	ROI‐average RMSE mean (a.u.)			ROI‐average RMSE SD (a.u.)		
	Standard linear	CASPR	Cardiac triggered sampling	Standard linear	CASPR	Cardiac triggered sampling
Brainstem	3.61 [3.27, 3.97]	3.51 [3.29, 3.81]	3.88 [3.46, 3.97]	0.88 [0.81, 1.05]	0.83 [0.69, 0.86]	0.93 [0.82, 1.04]
Cerebellum	3.73 [3.57, 3.83]	3.65 [3.43, 3.78]	3.81 [3.68, 4.05]	0.87 [0.76, 1.11]	0.78 [0.64, 0.90]	0.99 [0.70, 1.14]
Whole brain	2.90 [2.75, 3.01]	2.81 [2.70, 2.98]	2.93 [2.74, 3.11]	0.66 [0.61, 0.77]	0.60 [0.57, 0.63]	0.70 [0.59, 0.77]
Occipital lobe	2.09 [1.95, 2.42]	2.03 [1.86, 2.25]	2.11 [2.02, 2.47]	0.57 [0.50, 0.63]	0.45 [0.35, 0.50]	0.56 [0.46, 0.62]
Frontal lobe	1.71 [1.57, 1.82]	1.69 [1.60, 1.79]	1.71 [1.64, 1.85]	0.43 [0.40, 0.46]	0.38 [0.36, 0.42]	0.43 [0.38, 0.48]
Parietal lobe	1.44 [1.36, 1.58]	1.40 [1.36, 1.55]	1.46 [1.35, 1.63]	0.37 [0.31, 0.40]	0.33 [0.29, 0.35]	0.36 [0.29, 0.41]
Temporal lobe	2.34 [2.24, 2.53]	2.28 [2.16, 2.50]	2.41 [2.30, 2.56]	0.60 [0.57, 0.73]	0.53 [0.46, 0.57]	0.66 [0.53, 0.69]
Corticospinal tract	2.73 [2.40, 3.12]	2.63 [2.57, 2.94]	2.81 [2.59, 3.21]	0.64 [0.61, 0.75]	0.62 [0.57, 0.66]	0.70 [0.58, 0.78]
Inferior longitudinal fasciculus	2.25 [2.02, 2.64]	2.14 [2.12, 2.52]	2.27 [2.09, 2.68]	0.57 [0.47, 0.79]	0.51 [0.45, 0.57]	0.59 [0.50, 0.64]
Optic radiations	1.82 [1.62, 2.17]	1.76 [1.67, 2.00]	1.84 [1.70, 1.99]	0.46 [0.31, 0.61]	0.40 [0.33, 0.44]	0.48 [0.34, 0.50]

**FIGURE 5:**
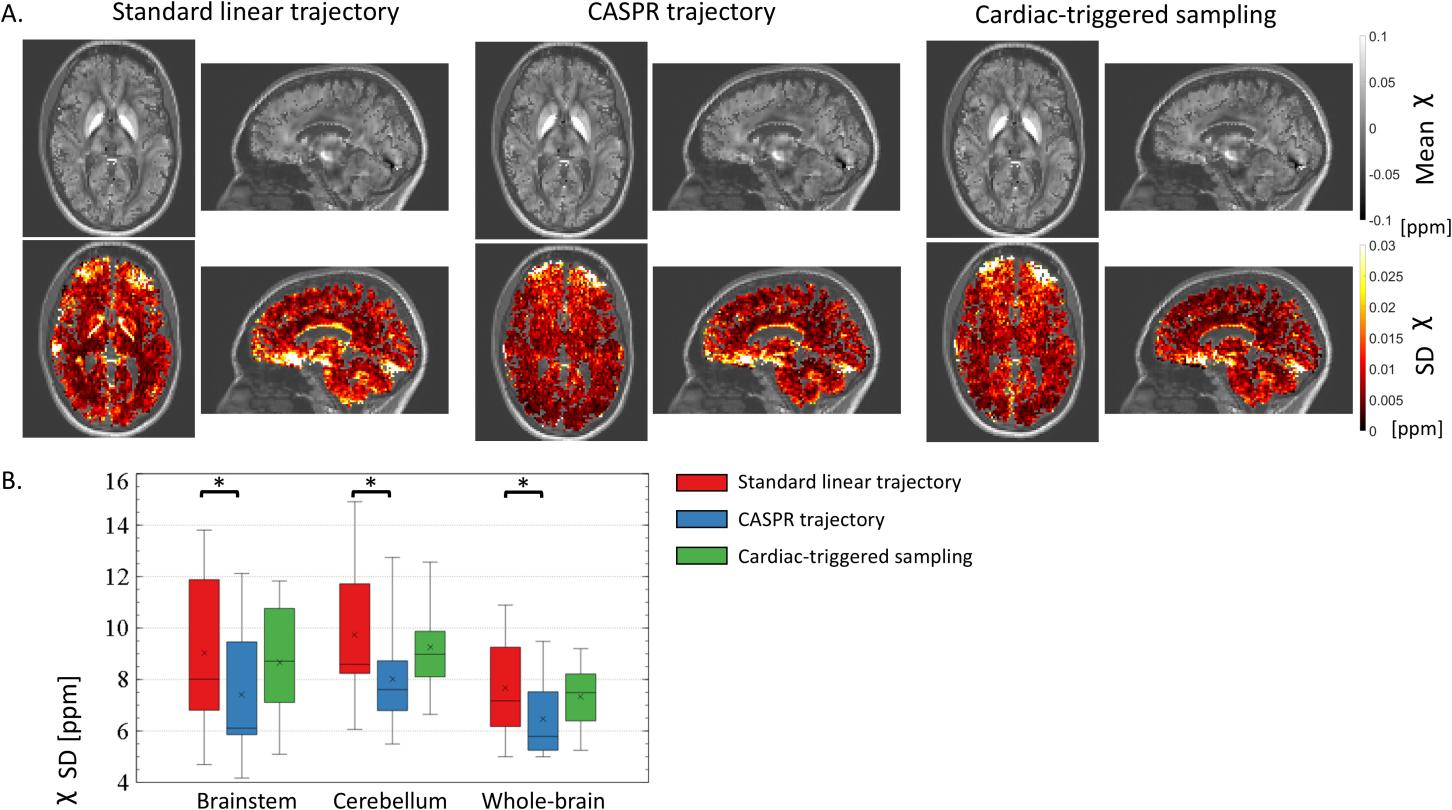
(A) Example maps of the mean and standard deviation (SD) of χ across repetitions, computed from data acquired with the standard linear trajectory, CASPR trajectory and cardiac triggered sampling. To improve visibility, the presented maps were masked using a whole‐brain mask including grey and white matter voxels (see Section 2.4.2) and overlaid on the MPRAGE image. (B) Regional estimates of the SD of χ across repetitions in the brainstem, cerebellum and whole brain, across all 10 healthy volunteers.

The maps of the mean and SD of χ across repetitions display the same spatial features between the three sampling strategies (Figure 5A). With the CASPR trajectory, the SD of the χ estimates across repetitions is lower than that of the standard linear trajectory by 19%/18%/16% in the brainstem/cerebellum/whole‐brain (Figure 5B, *p* ≤ 0.014). A reduction of the SD of χ is present across all brain regions, reaching 25%–30% in inferior brain regions such as the occipital and temporal lobes (Table 3). The smallest reduction of the SD of χ was ~15% in the grey matter of the frontal lobe. With the cardiac triggering, the SD of the χ estimates across repetitions is lower by 4%/5%/4% (Figure 5B, *p* ≥ 0.37).

**TABLE 3:** ROI‐average mean and variability of χ estimates across repetitions (median [interquartile range]), computed from data acquired with the standard linear trajectory, CASPR trajectory and cardiac triggered sampling.

	ROI‐average χ mean [ppm] × 10^3^			ROI‐average χ SD [ppm] × 10^3^		
	Standard linear	CASPR	Cardiac triggered sampling	Standard linear	CASPR	Cardiac triggered sampling
Brainstem	−3.59 [−7.88, −2.01]	−3.94 [−9.79, −1.49]	−3.94 [−9.79, −1.49]	8.01 [6.74, 11.96]	6.11 [5.79, 10.10]	8.71 [6.75, 10.95]
Cerebellum	−11.70 [−13.12, −10.66]	−11.59 [−12.48, −11.16]	−11.68 [−12.78, −11.16]	8.59 [8.23, 11.83]	7.61 [6.76, 8.95]	8.98 [7.98, 10.04]
Whole brain	−2.44 [−2.91, −1.99]	−2.42 [−3.03, −1.89]	−2.33 [−3.00, −1.80]	7.17 [6.03, 9.44]	5.79 [5.20, 7.71]	7.49 [6.32, 8.34]
Occipital lobe	6.89 [6.63, 7.46]	7.21 [6.73, 8.05]	6.78 [5.99, 7.35]	6.61 [5.19, 8.35]	4.47 [4.12, 5.17]	5.85 [5.30, 7.63]
Frontal lobe	2.82 [2.31, 3.04]	2.77 [2.43, 3.15]	2.65 [1.67, 3.21]	5.19 [4.92, 7.32]	4.50 [3.60, 6.04]	5.66 [4.79, 6.61]
Parietal lobe	3.87 [1.44, 4.46]	3.79 [1.21, 4.77]	3.56 [1.58, 4.84]	5.02 [3.72, 6.85]	3.61 [3.23, 4.95]	4.92 [4.22, 6.36]
Temporal lobe	5.02 [3.87, 8.33]	5.12 [3.59, 8.20]	5.14 [4.36, 7.99]	6.70 [4.87, 7.67]	4.61 [3.40, 5.54]	6.46 [4.99, 8.01]
Corticospinal tract	−16.71 [−17.78, −12.92]	−17.21 [−18.45, −12.48]	−16.04 [−18.41, −13.61]	5.12 [4.19, 6.74]	4.00 [3.31, 4.77]	5.04 [4.50, 6.79]
Inferior longitudinal fasciculus	−8.19 [−10.32, −5.62]	−8.19 [−11.28, −6.30]	−7.69 [−8.92, −5.97]	5.60 [3.73, 5.93]	3.66 [3.11, 4.60]	5.16 [4.14, 6.14]
Optic radiations	−13.17 [−14.62, −11.81]	−13.60 [−14.51, −12.28]	−12.94 [−14.45, −11.17]	4.84 [2.93, 6.71]	3.24 [2.61, 3.68]	4.61 [3.10, 5.86]

**FIGURE 6:**
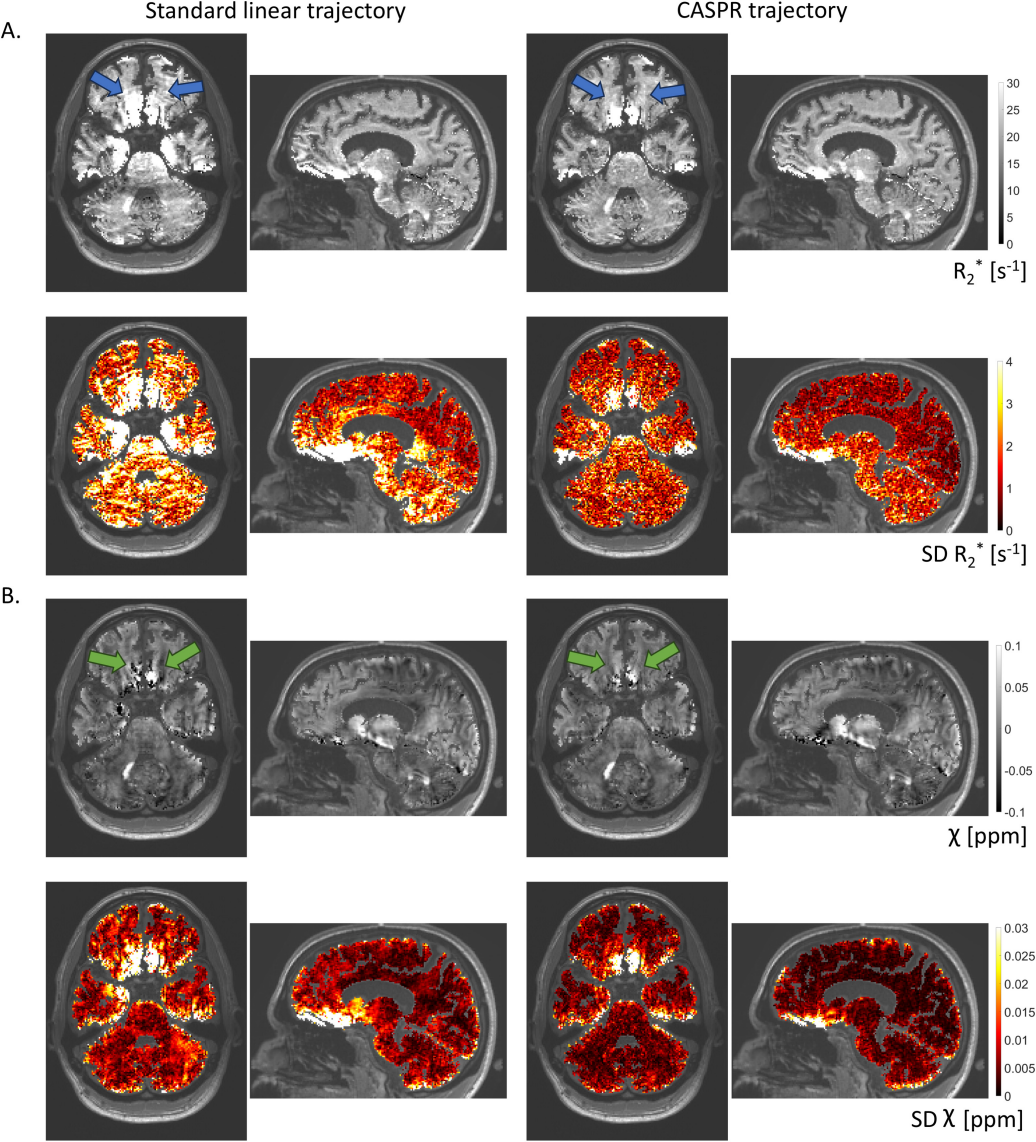
Maps of *R*_2_* and χ computed from higher resolution data acquired with the standard linear and CASPR trajectories. (A) *R*_2_* map from one repetition and standard deviation (SD) of *R*_2_* across repetitions. (B) χ map from one repetition and SD of χ across repetitions. Lower SDs can be observed throughout the brain for the CASPR trajectory. The blue and green arrows highlight coherent aliasing along the anterior–posterior direction, observable in the data from the standard linear trajectory but absent in the data from the CASPR trajectory. To improve visibility, the presented maps were masked using a whole‐brain mask including grey and white matter voxels (see Section 2.4.2) and overlaid on the MPRAGE image.

The high‐resolution data acquired with the standard linear trajectory show a clear pattern of increased variability across repetitions in inferior brain regions (Figure 6). In particular, maps of the SD of *R*_2_* show aliasing along the anterior–posterior direction of cardiac‐induced noise originally located, for example, in the Circle of Willis. This aliasing is not present in the data acquired with the CASPR trajectory. The SD of *R*_2_* across repetitions is lower by 31%/36%/36% in the brainstem/cerebellum/whole‐brain with the CASPR trajectory than the standard linear trajectory (Figure 6A). The SD of the χ estimates is lower by 27%/26%/32% with the CASPR trajectory than the standard linear trajectory in the brainstem/cerebellum/whole‐brain (Figure 6B). The reduction of the SD of *R*_2_* and χ maps across repetitions is larger than the $\sqrt{1.29}\sim 13.6\%$ reduction expected from the 29% of additional samples of CASPR trajectory if thermal noise dominated signal variance. *R*_2_* maps computed from data acquired with the standard linear trajectory also show aliasing of cardiac‐induced noise along the anterior–posterior direction (Figure 6A, blue arrows). With the CASPR trajectory, this aliasing is not present, and the delineation of the white‐grey matter is improved. The χ data acquired with linear trajectory show large streaking artefacts [56] around the Circle of Willis that are strongly reduced with CASPR trajectory (Figure 6B, green arrows).

## 4 Discussion

In this work, we proposed two new sampling strategies that aim to reduce the level of cardiac‐induced noise in brain maps of *R*_2_* and magnetic susceptibility. These strategies were derived from a recent characterization of cardiac‐induced noise in brain multiecho GRE data [27]. The first sampling strategy acquires a specific number of averages at each k‐space location, determined from the local level of cardiac‐induced noise. The second sampling strategy uses cardiac triggering to acquire the most sensitive region of k‐space in the part of the cardiac cycle where MRI signal is most stable. Both sampling strategies primarily target the k‐space centre, which contains most of the cardiac‐induced noise [27]. The ability of both proposed sampling strategies to reduce cardiac‐induced noise was assessed from estimates of the transverse relaxation rate (*R*_2_*), fitting residuals of *R*_2_* (RMSE) and magnetic susceptibility (χ).

With the standard linear trajectory, the timing of data acquisition within the cardiac period varies across repetitions, enhancing the variability of the *R*_2_* and χ estimates [25, 27]. The variability of *R*_2_* maps was 1.8–2.2 s^−1^ across repetitions, higher than the variability of *R*_2_* across the cardiac cycle in the original characterization of cardiac‐induced noise (0.8–1.4 s^−1^) [27]. However, this variability includes contributions from other noise sources such as thermal noise and respiration. Additionally, the current data displays spatial aliasing along the slow phase‐encoding direction (anterior–posterior in Figure 3A), leading to increased variability away from the source of cardiac‐induced noise. This aliasing was not present in the original characterization of cardiac‐induced noise because the k‐space data were binned according to its cardiac phase before image reconstruction [27].

The CASPR trajectory reduces the variability of *R*_2_* maps across repetitions by 20%–36% across the different regions, with inferior regions showing the strongest decrease. The variability of RMSE and χ maps is reduced by 9%–21% and 15%–30%. These reductions are stronger than those expected from the higher number of samples required with CASPR, if thermal noise dominates signal variance. This underlines the benefit of a strategy that targets the centre of k‐space, where most of the cardiac‐induced noise is located. However, the reduction of the variability of *R*_2_* and RMSE with CASPR is lower than the fraction of their variability attributed to cardiac pulsation in the original study (~35% and ~29% respectively) [27]. This is consistent with the lower impact of physiological noise in the high‐resolution data presented here, although the CASPR trajectory may have allowed for the correction of other types of physiological noise such as respiration [61].

Cardiac triggering was not effective at reducing the variability of *R*_2_*, χ and RMSE estimates across repetitions. We conducted further analyses to investigate the origin of this comparably poor performance and several contributing factors were identified (Supplementary Material S3). The number of peaks estimated from the pulse oximeter data in real time during MRI data acquisition was compared with that estimated offline using a Scholkmann algorithm optimized to avoid the identification of the dicrotic notch as a peak [62]. In two participants, the difference in the number of peaks reached 40%. In these instances, cardiac triggering would have further reduced the variability of the data if the Scholkmann algorithm had been used inline. However, two participants showed a strong increase in the SD of *R*_2_* and χ with cardiac triggering, although the number of dicrotic notches identified as peaks during data acquisition was low. This increase was due to poor image quality in isolated repetitions of data acquisition and may have been the result of other factors such as head motion. The number of cardiac triggering lines was set to 2, deemed sufficient to have a reliable and efficient synchronization (Figure 2). More cardiac triggering lines could have led to a better synchronization, at the cost of a longer scan time. However, finger pulse oximetry is inherently limited by the Pulse Arrival Time—the variable delay between ventricular contraction and the peripheral pressure wave—which introduces temporal jitter [63]. While ECG‐based triggering was not available for this specific scanner and pulse sequence, it would provide a near‐instantaneous electrical trigger (R‐wave) with the superior temporal precision required to align acquisition with the cardiac quiescent phase [64]. Moreover, even under the assumption that the synchronization was ideal, it is possible that changes in cardiac‐induced noise between heartbeats may lead to signal instabilities sufficient to counteract the intended benefits of cardiac triggering.

One limitation of CASPR trajectories is that they can induce stronger eddy current artefacts than their linear counterparts, due to the faster traversal of k‐space along the two‐phase encoding directions [65, 66]. Eddy currents can be mitigated by reducing the amplitude or slew rate of the encoding gradients, increasing the TR, or acquiring more points on each spiral arm to reduce the k‐space distance between consecutive points. Moreover, scanner heating leads to a drift of the main magnetic field that can degrade the quality of data acquired with CASPR trajectories. This effect can be corrected using phase navigator data acquired throughout the scans [67].

Here, we opted for CASPR trajectories to control for the k‐space sampling density in the 2D plane of the phase‐encoding directions. The readout direction was not considered because the readout time is typically shorter than the period of physiological signal fluctuations. Alternative noncartesian 3D trajectories would allow us to extend this work to the readout direction. 3D spiral‐UTE trajectories could be a suitable choice although long curved readouts might result in long echo spacings [68]. Conversely, 3D radial trajectories allow a fast multiecho acquisition with a sampling density that decreases with the square of the k‐space centre distance [69]. Both of those trajectories require careful implementation and precise characterization of the scanner's gradient system. Furthermore, the reconstruction is not trivial and requires advanced reconstruction techniques [70].

The proposed CASPR trajectory is a passive averaging approach that does not target specifically cardiac‐induced noise and acts on other sources of physiological noise such as motion and breathing. CASPR trajectories render physiological noise spatially incoherent: Physiological noise does not remain localized at its source but spreads across the 2D plane of the phase‐encoding directions [71]. Numerous methods exist to filter out incoherent aliasing artefacts, creating an opportunity for further data quality improvement or higher acceleration factors [70, 72]. Advanced reconstruction strategies such as compressed sensing [70] or HD‐PROST [72] synergise well with CASPR trajectories, which can be used to perform an incoherent undersampling of the k‐space data. The spatial scrambling of physiological noise with CASPR does not take place with the ‘ISME’ approach, which focuses on the temporal dimension of the multiecho data alone [25]. Both approaches lead to comparable reductions of the variability of *R*_2_* and χ across repetitions. One noticeable difference is that CASPR reduces the error level on the *R*_2_* estimates (RMSE) by ~5% in inferior brain regions, while ISME increases the RMSE by ~15% [25]. We attribute this difference to the fact that with CASPR the k‐space centre data, which contains most of the cardiac‐induced noise, is acquired at multiple phases of the cardiac cycle, for all echo times of the data.

## 5 Conclusions

In this work, we evaluated two candidate strategies that aimed to reduce the level of cardiac‐induced noise in brain maps *R*_2_* and magnetic susceptibility (χ). Both sampling strategies were motivated from a previous characterization of cardiac‐induced noise in brain 3D multiecho GRE data [27]. The first sampling strategy relies on a CASPR trajectory to acquire a number of averages at each k‐space location that depends on the local level of cardiac‐induced noise. The second strategy uses cardiac triggering to synchronize data acquisition with cardiac pulsation in real time to acquire the most sensitive area of k‐space during the most stable part of the cardiac cycle.

Compared to standard linear trajectory, cardiac triggering did not efficiently reduce the variability of *R*_2_* and χ estimates across repetitions. Conversely, the CASPR trajectories reduces the variability of *R*_2_* and χ estimates across repetitions by 15%–30%, for an increase in scan time of 14%. The largest improvements in variability take place in inferior brain regions such as the brainstem and cerebellum. CASPR further improves the reproducibility of *R*_2_* and χ maps by reducing the aliasing of cardiac‐induced noise across the field of view, away from its original location [67, 70, 72].

## Author Contributions

**Quentin Raynaud:** conceptualization, methodology, investigation, formal analysis, writing – original draft, writing – review and editing. **Thomas Dardano:** investigation, formal analysis, conceptualization, writing – review and editing. **Rita Oliveira:** investigation, conceptualization, writing – review and editing. **Giulia Di Domenicantonio:** investigation, conceptualization, writing – review and editing. **Tobias Kober:** investigation, conceptualization, writing – review and editing. **Christopher W. Roy:** investigation, conceptualization, writing – review and editing. **Ruud B. van Heeswijk:** conceptualization, methodology, investigation, formal analysis, writing – review and editing, supervision, funding acquisition. **Antoine Lutti:** conceptualization, methodology, investigation, formal analysis, writing – original draft, writing – review and editing, supervision, project administration, funding acquisition.

## Ethics Statement

This study received approval from the local ethics committee, and all participants gave their written informed consent prior to participation.

## Funding

This work was supported by the Schweizerischer Nationalfonds zur Förderung der Wissenschaftlichen Forschung (320030_184784, CR00I5‐235940, 32003B_182615, CRSII5_202276) and the Fondation Roger de Spoelberch.

## Supporting information


**Figure S1:** Bland–Altman plots of the *R*_2_* (A) and χ (B) estimates obtained from the standard linear trajectory, CASPR trajectory and the cardiac triggered sampling.
**Figure S2:** (A) Example of pulse oximeter signal (blue plot). The first and second triggering lines are indicated with arrows, and the time points where data acquisition was suspended are highlighted with red circles. (B) Example distribution of the phase of the cardiac cycle during the acquisition of the k‐space data, for the standard linear trajectory (left) and cardiac‐triggered sampling (right, the cardiac triggering lines are shown in red). The data were acquired with a GRAPPA acceleration factor of 2 with 24 reference lines at the k‐space centre. The missing lines are shown in dark blue. (C) Mean (left) and standard deviation (right) of the phase of the cardiac cycle during data acquisition across repetitions and participants using the cardiac‐triggered sampling.
**Figure S3:** (A) Standard deviation of R_2_* and χ across repetitions, averaged over the whole‐brain, for the standard linear trajectory and the cardiac‐triggered sampling. (B) Percentage of extra peaks detected by the inline peak detection compared to the offline Scholkmann algorithm. (C) R_2_* maps computed from the individual repetition of data acquisition in participant 6. Repetition 3 is of poor quality and a large variation of R_2_* values can be seen around the brainstem (orange). (D) Cardiac phase at the time of acquisition of the k‐space data, for the corresponding four repetitions.

## Data Availability

One of the 5D datasets used for the optimization of the sampling strategies can be found here (DOI:10.5281/zenodo.7428605). The high‐resolution dataset is available online here (DOI:10.5281/zenodo.12685105).
