# Supplementary material for: Reducing Noise Induced by Cardiac Pulsatility in Brain Maps of R 2* and Magnetic Susceptibility Using Tailored k‐space Sampling
Source: NMR Biomed. 2026 May 10;39:e70305. doi: 10.1002/nbm.70305 (PMC13158452; doi:10.1002/nbm.70305)
Supplement: Supplementary file 1 — Figure S1: Bland–Altman plots of the R 2* (A) and χ (B) estimates obtained from the standard linear trajectory, CASPR trajectory and the cardiac triggered sampling. Figure S2: (A) Example of pulse oximeter signal (blue plot). The first and second triggering lines are indicated with arrows, and the time points where data acquisition was suspended are highlighted with red circles. (B) Example distribution of the phase of the cardiac cycle during the acquisition of the k‐space data, for the standard linear trajectory (left) and cardiac‐triggered sampling (right, the cardiac triggering lines are shown in red). The data were acquired with a GRAPPA acceleration factor of 2 with 24 reference lines at the k‐space centre. The missing lines are shown in dark blue. (C) Mean (left) and standard deviation (right) of the phase of the cardiac cycle during data acquisition across repetitions and participants using the cardiac‐triggered sampling. Figure S3: (A) Standard deviation of R2* and χ across repetitions, averaged over the whole‐brain, for the standard linear trajectory and the cardiac‐triggered sampling. (B) Percentage of extra peaks detected by the inline peak detection compared to the offline Scholkmann algorithm. (C) R2* maps computed from the individual repetition of data acquisition in participant 6. Repetition 3 is of poor quality and a large variation of R2* values can be seen around the brainstem (orange). (D) Cardiac phase at the time of acquisition of the k‐space data, for the corresponding four repetitions. [file NBM-39-e70305-s001.docx]

Reducing noise induced by cardiac pulsatility in brain maps of R_2_* and magnetic susceptibility using tailored k-space sampling

Quentin Raynaud^1^, Thomas Dardano^1^, Rita Oliveira^1^, Giulia Di Domenicantonio^1^, Tobias Kober^2,3,4^, Christopher W. Roy^2^, Ruud B. van Heeswijk^2*^, Antoine Lutti^1*^

*^1^Laboratory for Research in Neuroimaging, Department for Clinical Neuroscience, Lausanne University Hospital and University of Lausanne, Lausanne, Switzerland*

*^2^Department of Diagnostic and Interventional Radiology, Lausanne University Hospital and University of Lausanne, Lausanne, Switzerland*

*^3^Advanced Clinical Imaging Technology, Siemens Healthineers International AG, Lausanne, Switzerland*

*^4^LTS5, École Polytechnique Fédérale de Lausanne (EPFL), Lausanne, Switzerland*

*Antoine Lutti and Ruud B. van Heeswijk contributed equally to this work.

**Correspondence**

Antoine Lutti

Laboratory for Research in Neuroimaging, Department for Clinical Neuroscience, Lausanne University Hospital, Ch. de Mont-Paisible 16, CH-1011 Lausanne

Email: [antoine.lutti@chuv.ch](mailto:antoine.lutti@chuv.ch)

# Supplementary material

## Systematic differences in R_2_* and $\chi$ between acquisition strategies

Because the standard linear and CASPR trajectories are not synchronized with cardiac pulsation, the resulting estimates of R_2_* are computed from data acquired across the cardiac cycle. As a result, no systematic differences are expected between those two sampling trajectories. However, cardiac triggering enforces acquisition of the most sensitive k-space data during the 1^st^ quarter of the cardiac cycle (Figure 2B). Because systematic changes of the apparent R_2_* take place across the cardiac cycle,^1^ cardiac triggering may lead to a systematic bias of the R_2_* estimates. This also applies to the estimates of $\chi$. However the computation of $\chi$ requires subtraction by a reference value, taken here as the average across the whole brain.^2^ This computation step removes any global systematic bias in the data.

Figure S1 shows bland-altman plots of the R_2_* and $\chi$ estimates, from the brain voxels of all participants. For the CASPR trajectory and cardiac triggering, the mean deviation of R_2_* from the standard linear estimates is -0.15s^-1^ and -0.08s^-1^ respectively. The mean deviation of the $\chi$ estimates is -2.8∙10^-6^ ppm and 4.1∙10^-6^ ppm respectively.


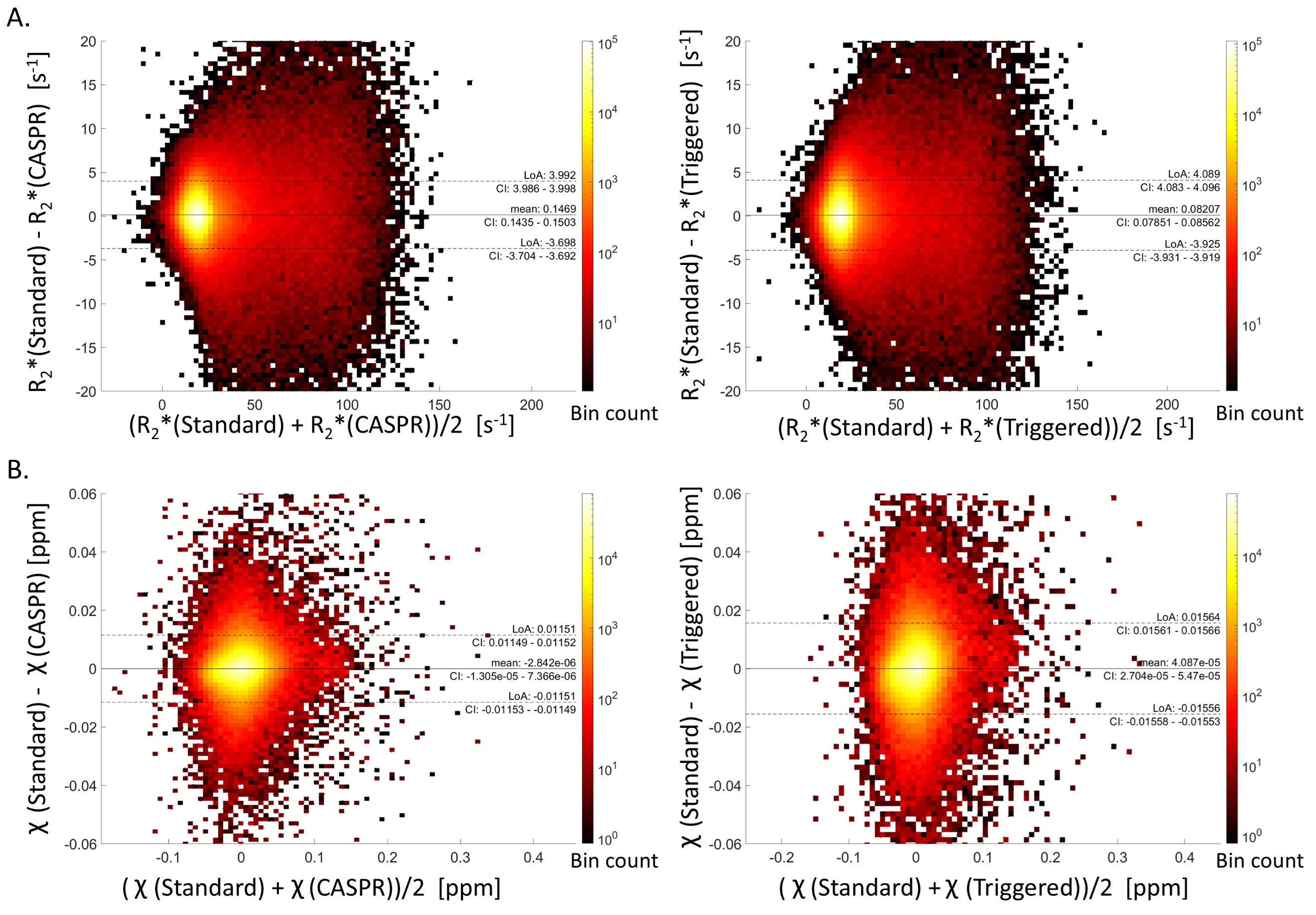


Figure S1: Bland–Altman plots of the R_2_* (A) and $\chi$ (B) estimates obtained from the standard linear trajectory, CASPR trajectory and the cardiac triggered sampling.

## Analysis of the cardiac-triggered sampling

Further analyses were performed to investigate the comparably poor performance of the proposed cardiac triggering approach.

Cardiac triggering was conducted from the detection of a peak in the pulse-oximeter data, in real time during data acquisition. The peaks were identified as local maxima with an amplitude above 2048 - the mean value of the pulse-oximeter signal. Upon visual inspection, the pulse-oximeter data was clean, and the peaks were accurately detected (figure S2A). Data acquisition was stopped when reaching the cardiac triggering lines ($k_{z}=k_{1}$ or $k_{z}=k_{2}$) and resumed after the detection of the subsequent peak in the pulse-oximeter data (Figure S2A).

Figure S2B shows the phase of the cardiac cycle at the time of acquisition of the k-space data. Along the fast phase-encoding direction, the time span between consecutive points (TR=40ms) is small compared to the cardiac period and the cardiac phase varies smoothly. For a standard linear trajectory (left), the phase of the cardiac cycle shows abrupt changes along the slow phase-encoding direction. As expected with cardiac triggering (right), the phase of the cardiac cycle is 0 (peak of the pulse wave detection) at the cardiac triggering lines, consistently along the slow encoding direction. The standard deviation of the phase of the cardiac cycle during the acquisition of the k-space centre data is below 0.6 rads (~10% of the cardiac phase) across repetitions and participants (Figure S2C).

Compared to that of the standard sampling, the standard deviation (SD) across repetitions of R_2_*- and $\chi$-mapping data acquired with cardiac triggering was highly variable between participants (Figure S3A). We compared the number of peaks detected inline during data acquisition and offline by a Scholkmann algorithm^3^ optimized to avoid the identification of the dicrotic notch as a peak (Figure S3B). The number of additional peaks detected inline varied greatly across participants, reaching ~40% for participant 1 and 5. For these participants, cardiac triggering with an inline implementation of the Scholkmann algorithm would have further reduced the SD of R_2_*- and $\chi$ across repetitions. However, for participants 6 and 10, the SD of R_2_* and $\chi$ strongly increased, although the number of dicrotic notches identified as peaks inline was small. This was due to one repetition of poor quality (repetition 3 for participant 6, see Figure S3C), although the k-space frequencies were consistently sampled in the expected range of cardiac phases (Figure S3D). These isolated repetitions of poor quality may therefore be the result of other factors such as head motion.

# Supplementary figures


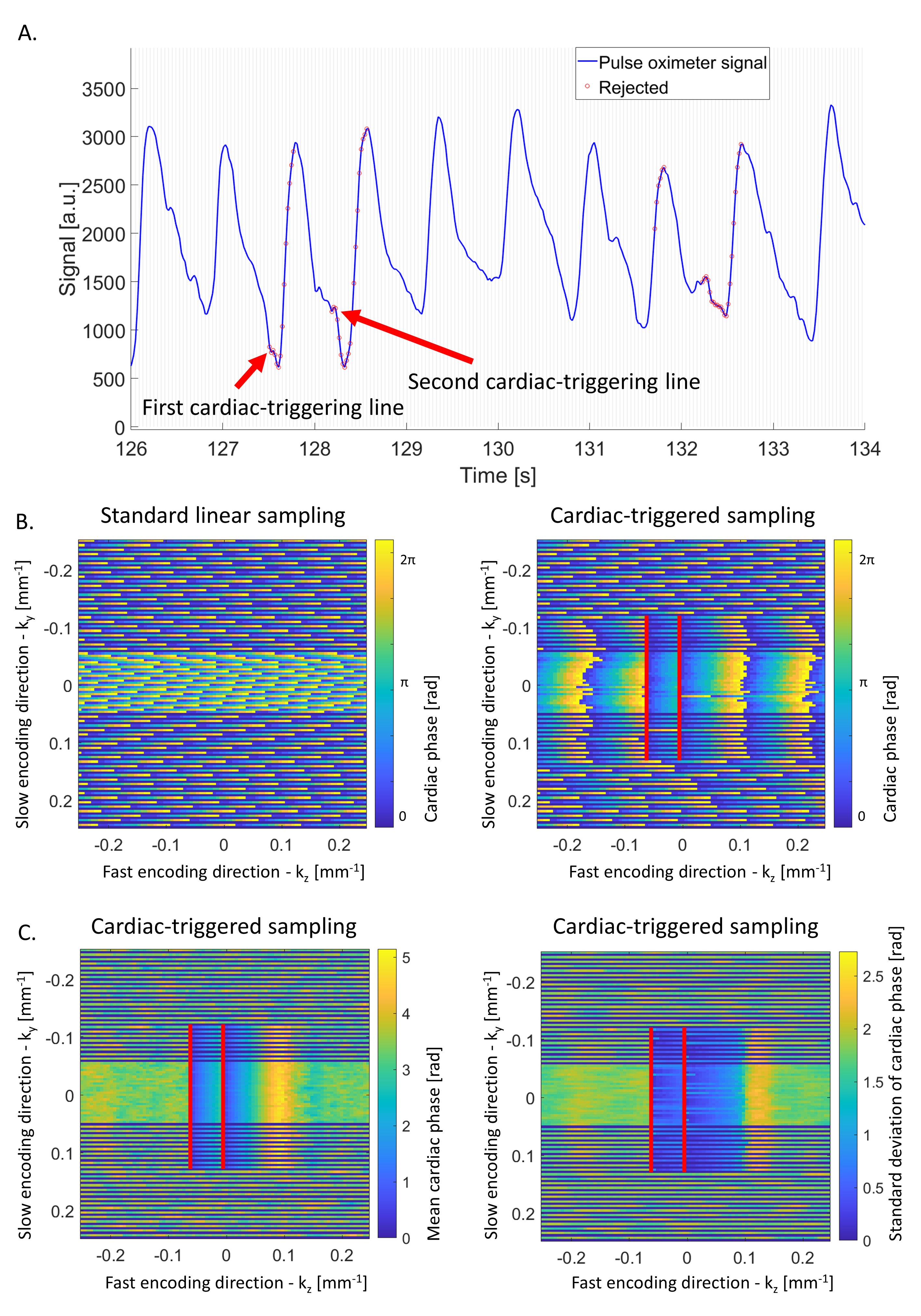


Figure S2: (A) Example of pulse oximeter signal (blue plot). The first and second triggering lines are indicated with arrows, and the time points where data acquisition was suspended are highlighted with red circles. (B) Example distribution of the phase of the cardiac cycle during the acquisition of the k-space data, for the standard linear trajectory (left) and cardiac-triggered sampling (right, the cardiac triggering lines are shown in red). The data were acquired with a GRAPPA acceleration factor of 2 with 24 reference lines at the k-space centre. The missing lines are shown in dark blue. (C) Mean (left) and standard deviation (right) of the phase of the cardiac cycle during data acquisition across repetitions and participants using the cardiac-triggered sampling.


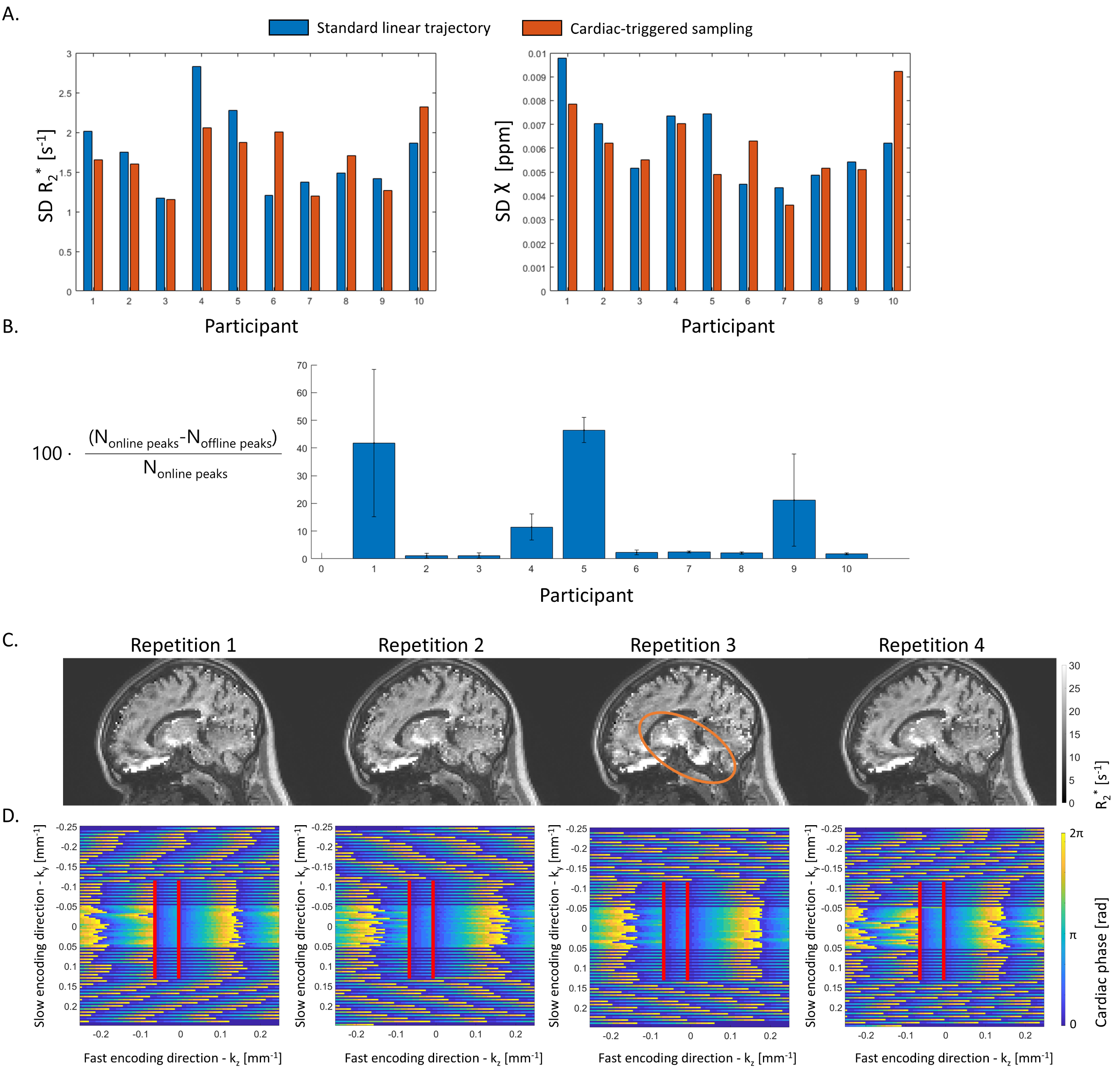


Figure S3: (A) Standard deviation of R_2_* and $\chi$ across repetitions, averaged over the whole-brain, for the standard linear trajectory and the cardiac-triggered sampling. (B) Percentage of extra peaks detected by the inline peak detection compared to the offline Scholkmann algorithm. (C) R_2_* maps computed from the individual repetition of data acquisition in participant 6. Repetition 3 is of poor quality and a large variation of R_2_* values can be seen around the brainstem (orange). (D) Cardiac phase at the time of acquisition of the k-space data, for the corresponding 4 repetitions.

# References:

1. Raynaud Q, Di Domenicantonio G, Yerly J, Dardano T, van Heeswijk RB, Lutti A. A characterization of cardiac-induced noise in R2* maps of the brain. *Magnetic Resonance in Medicine*. 2024;91(1):237-251. doi:10.1002/mrm.29853

2. Bilgic B, Costagli M, Chan KS, et al. Recommended implementation of quantitative susceptibility mapping for clinical research in the brain: A consensus of the ISMRM electro-magnetic tissue properties study group. *Magnetic Resonance in Medicine*. 2024;91(5):1834-1862. doi:10.1002/mrm.30006

3. Bishop SM, Ercole A. Multi-Scale Peak and Trough Detection Optimised for Periodic and Quasi-Periodic Neuroscience Data. In: Heldt T, ed. *Intracranial Pressure & Neuromonitoring XVI*. Vol 126. Acta Neurochirurgica Supplement. Springer International Publishing; 2018:189-195. doi:10.1007/978-3-319-65798-1_39
